# Plant-Derived Molecules α-Boswellic Acid Acetate, Praeruptorin-A, and Salvianolic Acid-B Have Age-Related Differential Effects in Young and Senescent Human Fibroblasts In Vitro

**DOI:** 10.3390/molecules25010141

**Published:** 2019-12-29

**Authors:** Anna Lewinska, Lakshman Sodagam, Dominika Bloniarz, Karsten Siems, Maciej Wnuk, Suresh I. S. Rattan

**Affiliations:** 1 Department of Biotechnology, University of Rzeszow, 35-310 Rzeszow, Poland; dominikabloniarz@outlook.com (D.B.); mawnuk@gmail.com (M.W.); 2 Laboratory of Cellular Ageing, Department of Molecular Biology and Genetics, Aarhus University, 8000 Aarhus C, Denmark; ls@mbg.au.dk; 3 AnalytiCon Discovery GmbH, 14473 Potsdam, Germany; k.siems@ac-discovery.com

**Keywords:** human fibroblasts, replicative age, hormesis, phytochemicals, oxidative stress response, autophagy, inflammation

## Abstract

Testing and screening of plant-derived molecules on normal human cells in vitro is a widely used approach for discovering their eventual health beneficial effects for human ageing and longevity. As little is known about age-associated differential effects of such molecules, here we report that young (<25% replicative lifespan completed) and near-senescent (>90% replicative lifespan completed) human skin fibroblasts exposed for 1–15 days to a wide range of concentrations (0.1–100 μM) of the three selected phytochemicals, namely α-boswellic acid acetate (ABC), praeruptorin-A (PTA), and salvianolic acid-B (SAB) had age-related differential effects. The parameters studied were the metabolic activity (MTT assay), cellular morphological phenotype, one-step growth characteristics, expression of genes involved in the cell cycle regulation and cytokine network genes, protein levels of p53, cytosolic superoxide dismutase (SOD1) and microtubule-associated protein 1A/1B-light chain 3 (LC3), and the extent of protein carbonylation and protein aggregation as a sign of oxidative stress. All three compounds showed biphasic hormetic dose response by stimulating cell growth, survival and metabolic activity at low doses (up to 1 μM), while showing inhibitory effects at high doses (>10 μM). Furthermore, the response of early passage young cells was different from that of the late passage near-senescent cells, especially with respect to the expression of cell cycle-related and inflammation-related genes. Such studies have importance with respect to the use of low doses of such molecules as health-promoting and/or ageing-interventions through the phenomenon of hormesis.

## 1. Introduction

Testing and screening natural compounds, extracted from plants, for their potential benefits for human health is one of the topical areas in preventive and therapeutic biomedicine. Most commonly, phytochemicals are claimed to be potentially health beneficial, such as being anti-cancer, anti-inflammatory, and anti-ageing [1,2,3,4,5,6]. Notwithstanding overhyped claims and empty promises made for their human applications, testing active components by using various experimental model systems is a valid and crucial first step for any future discoveries and development [7]. Identification of potential health beneficial effects of numerous polyphenols, both flavonoids (such as quercetin, catechins, genistein, etc.) and non-flavonoids (such as resveratrol, curcumin, caffeic acid, etc.), as well as ginsenosides, tocotrienols, and urolithins, is the result of such a strategy [8].

We have also utilized the above approach for screening phytochemicals by using the Hayflick model system of human cellular ageing in vitro [9], and have reported several ageing-modulatory effects of kinetin, zeatin, curcumin, rosmarinic acid (ROSM), ampelopsin (AMPEL), and amorfrutin-A (AMOR) [10,11,12,13]. Such a research and development strategy is also supported by various governmental and private funding agencies, including the European Union (EU), through research and innovation actions (RIA). Within one of these RIA titled “Understanding health, ageing and disease: determinants, risk factors and pathways”, is our project “Ageing with elegans” (AwE) with the aim of testing and screening natural and synthetic phytochemicals for their effects on ageing, longevity, and health of human cells in vitro [14].

**Figure 1:**
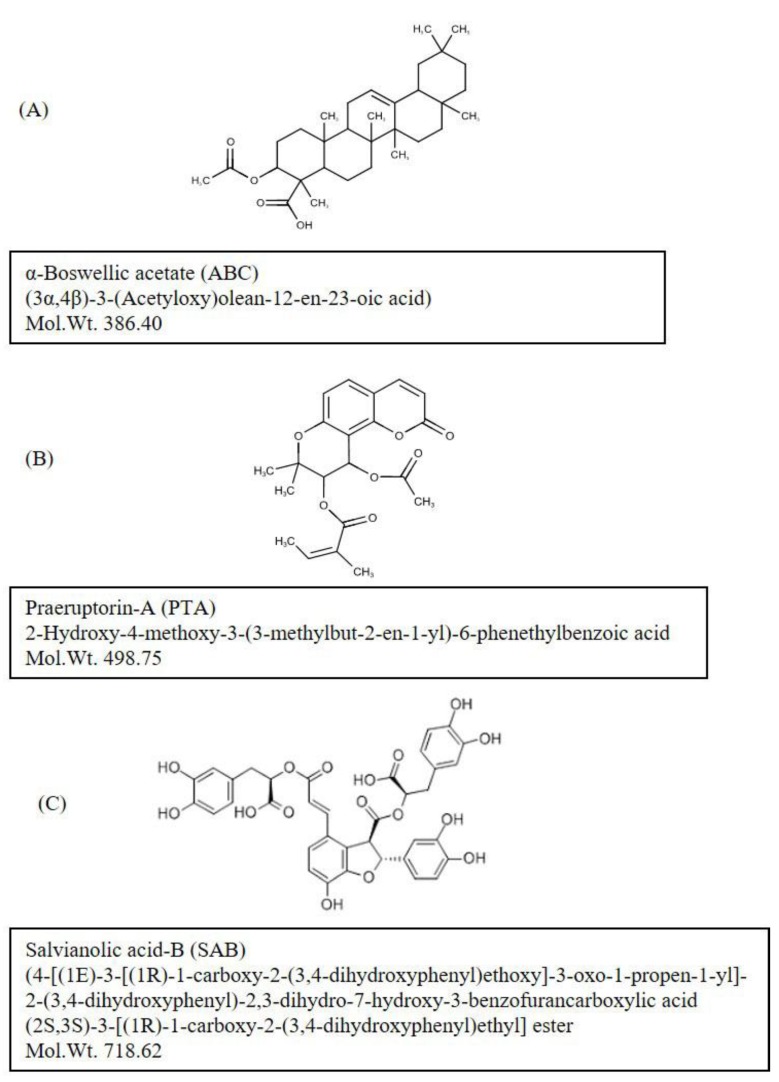
Structures of test compounds: (**A**): α-boswellic acid acetate (ABC); (**B**): Praeruptorin-A (PTA); and (**C**): Salvianolic acid-B (SAB). Further information on the identity and purity confirmation of compounds NMR and HPLC-MS-ELSD is available on file at the AnalytiCon Discovery, Germany.

Recently, we have published the results of our short- and long-term studies on the effects of ROSM, AMPEL, and AMOR on serially passaged human skin fibroblasts within the framework of the above RIA [13]. In continuation with the above RIA, we have now tested three other compounds, namely α-boswellic acid acetate (ABC), praeruptorin-A (PTA), and salvianolic acid-B (SAB) on human skin fibroblasts undergoing serial passaging in vitro. A brief description of the origin, chemical structures, and the reported biological effects of the test compounds is given below:

(i) ABC (Figure 1A) is an active ingredient extracted from the gum resin of the African myrrhe tree, *Commiphora myrrhe*, as well as from different species of the genus *Boswellia*, mainly *Boswellia serrata* and *Boswellia sacra*. The whitish to pale brown bark of the plant exudes a fragrant resin called Frankincense, an aromatic resin used in incense and perfumes [15,16]. Boswellic acids are pentacyclic triterpenes, and have been reported to have anti-inflammatory, anti-arthritic, analgesic, and anti-cancer effects [15,16,17,18]. ABC has also been reported to have anti-inflammatory effects on human fibroblasts and epithelial cells [19].

(ii) PTA (Figure 1B) [(9R,10S)-10-acetyloxy-8,8-dimethyl-2-oxo-9,10-dihydropyrano [2,3-f]chromen-9-yl](Z)-2-methylbut-2-enoate); is the main bioactive constituent of the *Angelica archangelica*, commonly known as wild celery. PTA has been used in traditional Chinese medicine for the treatment of cold, cough, and respiratory infections [20]. PTA has been shown to have anti-cancer effects against several types of cells in humans [21,22]. Further pharmacological studies indicate that PTA’s beneficial effects may be due to its anti-inflammatory effects involving the suppression of MMP-2 expression and ERK1/2 signaling [22,23,24,25]. 

(iii) SAB (Figure 1C) (4-[(1E)-3-[(1R)-1-carboxy-2-(3,4-dihydroxyphenyl) ethoxy] -3-oxo-1-propen-1-yl]-2-(3,4-dihydroxyphenyl)-2,3-dihydro-7-hydroxy-3-benzofurancarboxylic acid (2S,3S)- 3-[(1R)-1-carboxy-2-(3,4-dihydroxyphenyl)ethyl]ester), is a free radical scavenger extracted from *Salvia miltiorrhiza*, commonly known as the red sage, and from several other species of the *Lamiaceae*. SAB has been used for its cardioprotective [26], and chemopreventive effects for head and neck squamous cell cancer [27]. It has also been reported to reduce leukocyte-endothelial adherence, inhibit inflammation and metalloproteinase expression from aortic smooth muscle cells, and regulate immune function and certain intracellular kinase associated signaling pathways by competitively inhibiting the protein-protein interactions mediated by key binding domains [28]. SAB is an important ingredient in traditional Chinese medicine successfully used in testing and preventing age-related cardiovascular and cerebrovascular diseases and cancers [29].

Here we report the results of our investigations on comparing the effects of ABC, PTA, and SAB, tested individually, for their differential effects on early passage young human skin fibroblasts (<25% lifespan completed in vitro) versus late passage near senescent cells (>90% lifespan completed in vitro), treated for a shorter period (between 1 and 15 days). The parameters studied were the metabolic activity, cellular morphological phenotype, one-step growth characteristics, expression of genes involved in the cell cycle regulation and the cytokine network, protein levels of p53, cytosolic superoxide dismutase (SOD1) and microtubule-associated protein 1A/1B-light chain 3 (LC3), and the extent of protein carbonylation as a sign of oxidative stress. Our results show that the three compounds tested in this study have a typical biphasic hormetic dose response by stimulating cell growth, survival and metabolic activity at low doses, while showing inhibitory effects at high doses. Furthermore, the response of early passage young cells was different from that of the late passage near-senescent cells, especially with respect to the expression of cell cycle-related and inflammation-related genes.

## 2. Results and Discussion

### 2.1. Age-Related Differential Effects on Metabolic Activity and Cellular Growth

**Figure 2:**
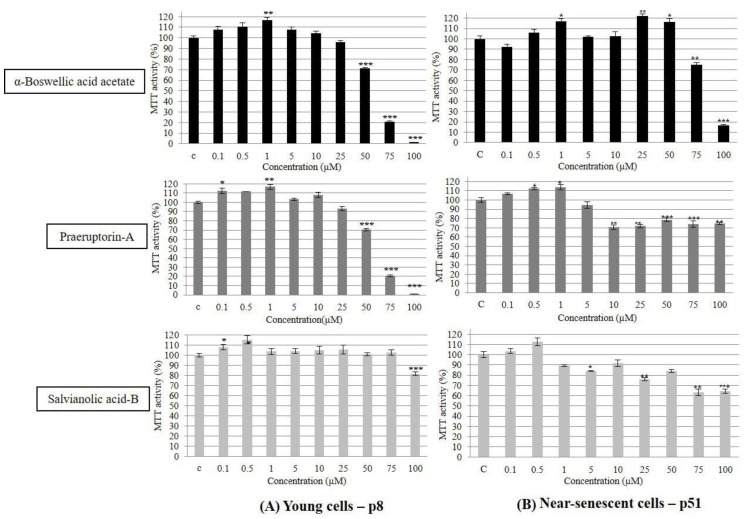
Dose response testing (between 0.1 and 100 μM) of ABC, PTA, and SAB on the metabolic activity and survival of early passage (p8, (**A**)) young and late passage (p51, (**B**)) near-senescent human skin fibroblasts (PCS cells), as determined by the metabolic activity (MTT assay). The bars indicate the ±SD; *n* = 6, in terms of independent wells; *** *p* < 0.001, ** *p* < 0.01, * *p* < 0.05, as determined by Student’s *t*-test.

Three compounds ABC, PTA and SAB were tested individually, in a thousand-fold concentration range (between 0.1 and 100 µM), on human skin fibroblasts (PCS cells). The results were mostly biphasic in response, as determined by MTT assay that measures both the survival and the metabolic activity of cells. The biphasic dose response is a well-known phenomenon of hormesis in which low doses of potentially toxic chemicals have opposite and potentially health beneficial effects [7,14]. Figure 2A shows that a 2-day exposure of young cells (p8) to any of the three compounds showed good tolerance up to about 10 µM concentration, followed by a decline in MTT activity reaching almost 100% toxicity at 100 µM, except for SAB. There were some differences among the three tested compounds. For example, while both ABC and PTA significantly increased the MTT activity up to 5 µM, SAB was stimulatory only up to 1 µM. Similarly, while both ABC and PTA showed dose-dependent inhibitory activity from 10 µM onwards, SAB became significantly inhibitory only at 100 µM, which was still much less inhibition as compared to ABC and PTA (Figure 2A).

The effects of ABC, PTA and SAB on near-senescent (p51) PCS cells were also biphasic to a large extent (Figure 2B). Whereas both ABC and PTA were significantly stimulatory up to 1 µM concentration, SAB peaked its stimulatory effect at 0.5 µM. After these concentrations, all three compounds showed inhibitory effects on MTT activity, but to a lesser extent as compared with the young cells. This observation is in line with the well-established fact that non-proliferating or slow-proliferating senescent cells often resist toxin-induced cell death [30,31,32]. It will be useful to further evaluate the effects of ABC, PTA and SAB on other metabolic parameters by using more sensitive methods.

**Figure 3:**
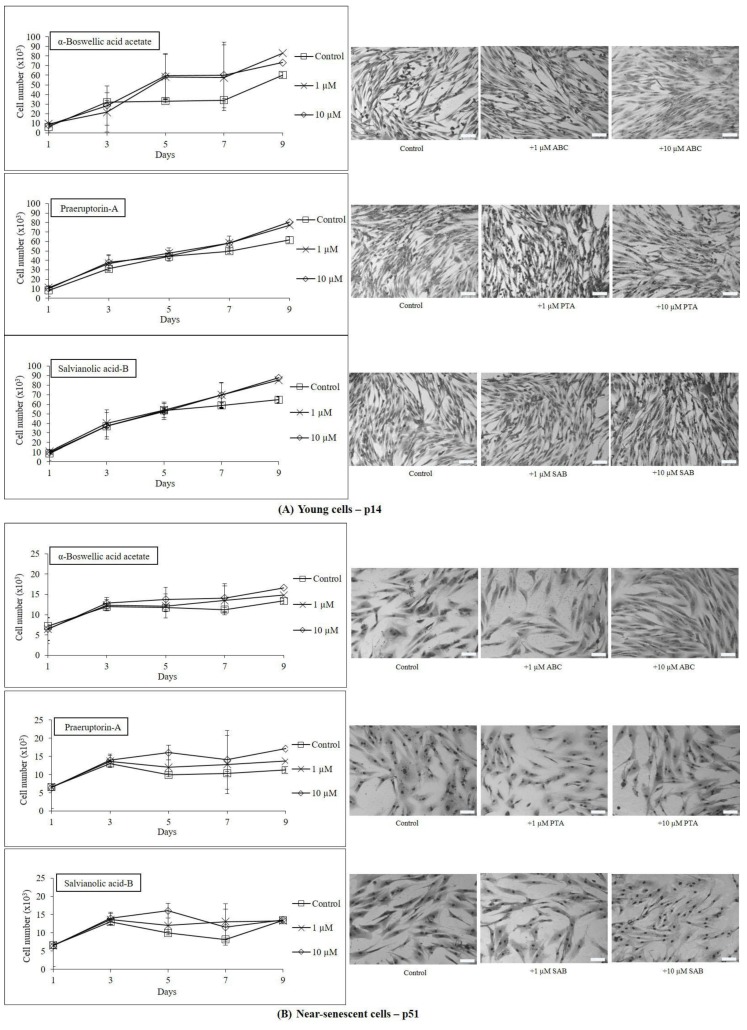
One-step growth curves and cell morphology of early passage young (p14, (**A**)) and late passage near-senescent (p51, (**B**)) PCS cells exposed to two concentrations (1 and 10 μM) of test compounds over a period of 9 days; *n* = 3; ±SEM; and photographs of Giemsa stained cells at microscopic magnification using 10× objective; scale bar: 100 µm.

On the basis of the MTT results mentioned above, two concentrations (1 and 10 µM) of test compounds were selected for their effects on short term growth and morphology (up to 9 days). Figure 3 shows that all three compounds (ABC, PTA, and SAB) were growth supportive or slightly stimulatory for both early passage young (Figure 3A) and near-senescent cells (Figure 3B), but the differences were not statistically significant. This effect was further documented by showing the microphotographs of Giemsa-stained young and near-senescent PCS cells which maintained their morphology or looked thinner and elongated in the case of near-senescent cells (Figure 3B).

**Figure 4:**
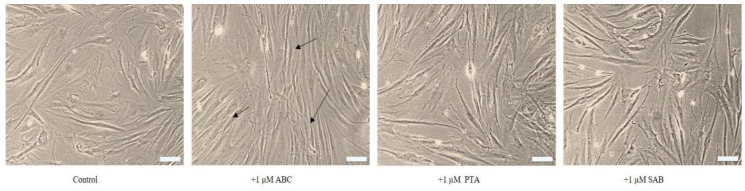
Effect of test chemicals on the morphology of senescent PCS cells (p58) after 15 days of exposure. Phase contrast photographs of live cells, microscopic magnification using 10× objective; scale bar: 100 µm. Arrows indicate rejuvenating or morphological-reversion effects of the test compounds.

The possible rejuvenating or morphological-reversion effects of the test compounds were further tested on replicatively senescent cells at p58. Figure 4 shows that whereas control cells had typical senescent morphology in terms of being large, flat, irregularly arranged, and full of debris, cells treated with 1 μM ABC, PTA, or SAB were somewhat morphologically rejuvenated. This effect was more apparent in case of ABC-treated cells where a majority of the cells became elongated and rearranged in regular arrays (Figure 4; arrows). In this pilot study the so-called rejuvenation is only inferred from morphological observations, and would require further molecular determinations, such as epigenetic status including DNA methylation and telomere length as markers of ageing [9,32].

### 2.2. Cell cycle progression and the expression of proliferation-related genes

**Figure 5:**
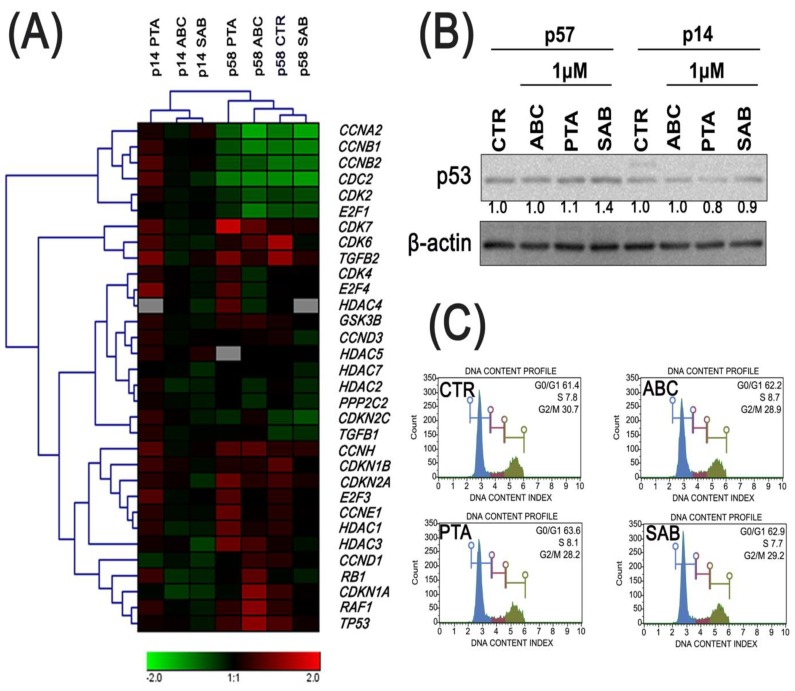
ABC, PTA, or SAB-induced changes in the cell cycle and cell cycle regulators in PCS cells at early (p14) and late passages (p57/58) after exposure for 24 h. (**A**) The expression profile of selected genes involved in the regulation of cell cycle. The levels of gene expression presented as the relative log10 values compared to control conditions (cells at p14 without treatments) and normalized to *GAPDH* gene expression. A heat map generated from qRT-PCR data is shown. (**B**) Western blot analysis of the levels of p53 cell cycle inhibitor. Anti-β-actin antibody was used as a loading control. The data represent the relative density normalized to β-actin. (**C**) DNA content-based analysis of cell cycle of late passage cells was conducted using flow cytometry. Representative histograms are presented.

**Figure 6:**
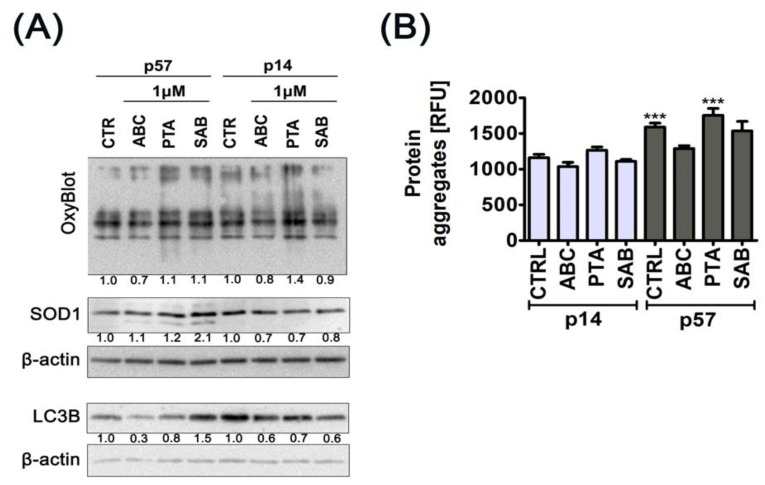
ABC-, PTA-, or SAB-induced changes in the levels of oxidative protein damage (protein carbonylation), SOD1 and LC3B level (**A**), and in the formation of protein aggregates (**B**) in PCS cells at early (p14) and late passages (p57). (**A**) Protein carbonylation was revealed using 2,4-dinitrophenylhydrazine (DNPH) derivatization and anti-DNP antibody. Western blot analysis of the levels of SOD1 and LC3B. Anti-β-actin antibody was used as a loading control. The data represent the relative density normalized to β-actin. (**B**) Protein aggregation was estimated by using PROTEOSTAT^®^ Protein Aggregation kit according to the manufacturer’s instructions (Enzo Life Sciences, Inc., Farmingdale, NY, USA). Protein aggregates are presented as relative fluorescence units (RFU). Bars indicate SD, *n* = 3, *** *p* < 0.001 compared with control at early passage (ANOVA and Dunnett’s a posteriori test).

**Figure 7:**
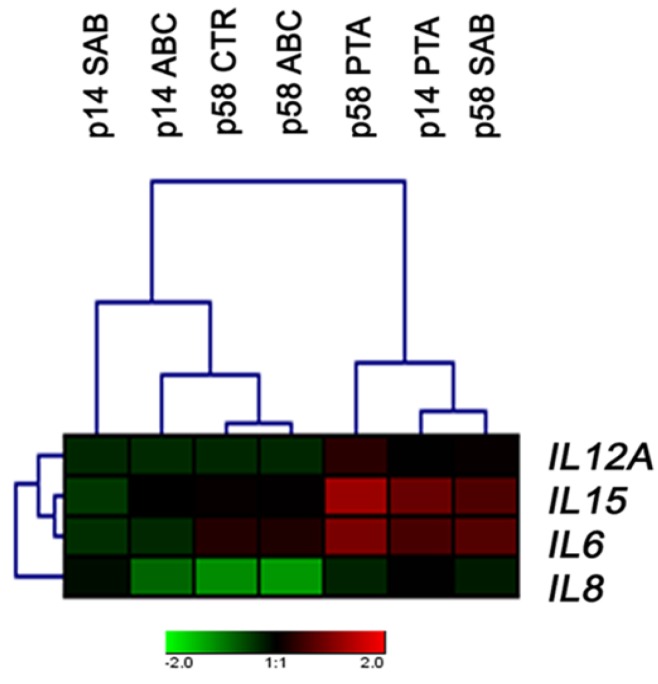
ABC-, PTA-, or SAB-induced changes in the expression of selected interleukin genes in PCS cells at early (p14) and late (p58) passages. A heat map generated from qRT-PCR data is shown. Hierarchical clustering was created using Genesis 1.7.7 software (Graz University of Technology, Graz, Austria).

Age-associated effects of the three test compounds in terms of changes in the progression of cell cycle, expression patterns of genes involved in the regulation of cell cycle and inflammatory responses, susceptibility to oxidative protein damage and the expression of antioxidant enzyme SOD1 and a biomarker of autophagy LC3BII are presented in Figure 5, Figure 6 and Figure 7.

The cells at late passage were characterized by decreased expression of *CCNA2* (cyclin A2), *CCNB1* (cyclin B1), *CCNB2* (cyclin B2), *CDC2* (cyclin dependent kinase 1), *CDK2* (cyclin dependent kinase 2), and *E2F1* (E2F transcription factor 1) genes, and increased expression of *CCNH* (cyclin H), *CDKN1B* (p27^Kip1^), *CDKN2A* (p16^INK4A^), *E2F3* (E2F transcription factor 3), *CCNE1*(cyclin E1), *HDAC1* (histone deacetylase 1), *CCND1* (cyclin D1), *CDKN1A* (p21), *RAF1* (raf-1 proto-oncogene, serine/threonine kinase) and *TP53* (p53) genes as compared to cells at early passage (Figure 5A).

Treatment of early passage cells with ABC resulted in decreased expression of the *CCNH*, *CDKN1B*, *CDKN2A*, *E2F3*, *CCNE1*, *HDAC1*, *CCND1* (cyclin D1), *CDKN1A*, *CDK7* (cyclin dependent kinase 7), and *TGFB2* (transforming growth factor beta 2) genes as well as a slight increase in *CDKN2C* (p18) and *CDKN1A* gene expression compared to untreated control cells at late passage (Figure 5A).

In the case of 24 h PTA-treatment of cells at early passage, there was an increase in the expression of majority of genes involved in cell cycle regulation, including *RB1* (RB transcriptional co-repressor 1), *CDKN1B*, *CDKN2A* and *CDKN2C* genes. SAB-mediated changes in the expression profiles between cells at early and late passages were subtle, except of decreased expression of *TP53*, *HDAC1,* and *CDKN2A* genes, and increased expression of *HDAC5* (histone deacetylase 5) gene compared to untreated control cells at early passage (Figure 5A).

In the case of SAB-treatment, compared to untreated control cells at late passage, SAB caused a slight decrease in the expression of some genes such as *RB1*, *HDAC3* (histone deacetylase 3), *CCND3*, *CDK6* and *TGFB2* (Figure 5A). Furthermore, in SAB-treated cells at late passage an increase of 40% in the levels of p53 was observed as compared to untreated control (Figure 5B). An analysis of the cell cycle in the late passage cells did not show significant changes in the G1, S, and G2/M phases in any of the phytochemical-treated cells as compared to the control conditions (Figure 5C).

### 2.3. Oxidative Protein Damage, Protein Aggregation and Autophagy

It is well known that plant-derived natural substances may affect the intracellular redox milieu [33]. Therefore, we determined the levels of ABC-, PTA-, and SAB-mediated oxidative protein damage, namely protein carbonylation, expression of antioxidant enzyme superoxide dismutase SOD1 (Figure 6A), and the formation of protein aggregates as a consequence of protein damage (Figure 6B).

The effects elicited by test compounds were slight to moderate, and were age-dependent. For example, PTA increased protein carbonylation of cells at early passage, whereas ABC decreased the levels of protein carbonylation and SAB caused a minor decrease and increase in protein carbonylation of cells at early and late passages, respectively (Figure 6A). The expression of SOD1 was increased only upon treatment of cells at late passage with SAB that was also accompanied by elevated expression of LC3BII, a biomarker of autophagy (Figure 6A). This suggests that SAB is able to induce autophagy in cells at late passage (Figure 6A). The most pronounced protein carbonylation after PTA treatment (Figure 6A) was also accompanied by the formation of protein aggregates in cells at late passage (Figure 6B).

In contrast, a decrease of LC3BII levels was observed in ABC-, PTA-, and SAB-treated cells at early passage (Figure 6A). SAB has been already established as an inducer of autophagy in different biological settings [34,35,36,37,38]. SAB-induced autophagy plays a pro-death role and contributed to the cell death of colorectal cancer cell lines through the suppression of AKT/mTOR pathway [35]. AKT/mTOR signaling pathway is also involved in SAB-induced autophagy and apoptosis in hepatocellular carcinoma cells [36]. SAB also promotes cardioprotective effects in rat acute myocardial infarction model by the induction of expression of antioxidant enzyme SOD1, anti-apoptotic protein Bcl-2, and autophagy proteins, namely LC3-II and Beclin 1 [34]. SAB also protects human umbilical vein endothelial cells (HUVEC) against hydrogen peroxide-induced oxidative stress by promoting autophagy via the activation of AMPK pathway and the downregulation of mTOR pathway [37,38]. SAB also limits alpha-synuclein aggregation and induces chaperone-mediated autophagy in cellular models of alpha-synuclein aggregation [38]. 

### 2.4. The Expression of Cytokine Genes

SAB is known to exhibit anti-inflammatory properties that prevent astroglia- and microglia-mediated neuroinflammation in BAC-alpha-syn-GFP transgenic mice [39]. SAB also protects against oxidative stress and inflammation during lipopolysaccharide (LPS)-induced acute lung injury (ALI) in rats by modulating the levels of malondialdehyde, superoxide dismutase, catalase, glutathione peroxidase, tumor necrosis factor-alpha, and interleukin 6 (IL6) [39]. We have therefore evaluated the compound-mediated effects on the expression of selected genes involved in inflammatory responses, and significant changes in the expression profiles of IL12A, IL15, IL8, and IL6 were noticed (Figure 7). 

SAB promoted an increase in the levels of IL15 and IL6 in cells at late passage, but in cells at early passage, SAB decreased the expression of IL15 and IL6 (Figure 7). In comparison, PTA increased the expression of IL15 and IL6 in cells at both passages. Furthermore, a decrease in the levels of IL6 was observed in ABC-treated cells at early passage (Figure 7). Indeed, anti-inflammatory activity of ABC has also been reported earlier [40]. For example, ABC inhibits NF-kappaB signaling and decreased TNF-alpha expression by direct interaction with IkappaB kinases in LPS-stimulated human peripheral monocytes [40].

**Figure 8:**
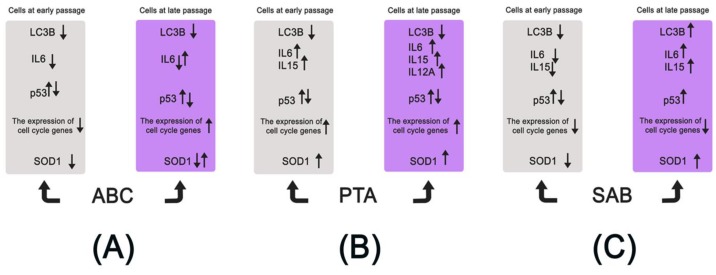
Replicative age-related effects of three test phytochemicals, ABC (**A**), PTA (**B**), and SAB (**C**) on human young (p14) and near senescent (p58) PCS cells in vitro. Several parameters were considered, namely protein levels of SOD1 (oxidative stress response), LC3B (autophagy), p53 (cell cycle regulation), and mRNA levels of selected cell cycle regulators (cell cycle progression) and interleukins (inflammatory response). In general, cells at early and late passages responded differently, especially in the case of SAB, which, when added at an early passage, caused a decrease in IL6 and IL15 levels and acted as an anti-inflammatory agent, whereas at late passage, it promoted adaptive stress response that was based on SAB-induced expression of SOD1 and autophagy.

A summarizing scheme presenting the pleiotropic and age-related differential action of ABC, PTA, and SAB in human skin fibroblasts at early and late passages is shown in Figure 8.

## 3. Materials and Methods

### 3.1. Phytochemicals

Detailed information about the extraction and purity analysis of compounds is available on file at the AnalytiCon Discovery, Germany. Briefly, ABC was isolated from *Commiphora myrrhe*, (collected in 2007 by Thomas Friedrich, Friedrich Nature Discovery, Euskirchen, Germany), by reverse phase chromatography (batch ID H-2052-C-13). NMR studies confirmed the identity and HPLC-MS the purity (100%; analyzed by HPLC-MS-ELSD at AnalytiCon Discovery). PTA was isolated from roots of *Angelica archangelica* (purchased in 2010 from Galke GmbH, Gittelde, Germany) by reverse phase chromatography (batch ID C-1171-E-14). NMR studies confirmed the identity and HPLC-MS the purity (99.8%; analyzed by HPLC-MS- ELSD at AnalytiCon Discovery). SAB was isolated from roots of *Salvia miltiorrhiza* (purchased in 2010 from Herbasin GmbH, Hilsdorf, Germany) by reverse phase chromatography (batch ID C-1173-C-02). NMR studies confirmed the identity and HPLC-MS the purity (87.5%; analyzed by HPLC-MS- ELSD at AnalytiCon Discovery).

Test compounds ABC, PTA, SAB were dissolved individually in DMSO making a stock solution of 1 mM each, having a maximum concentration of DMSO at 0.5% [13]. The stock solutions were stored at 4 °C until further use when appropriate volume from the individual stock solution was added to the complete culture medium in the flasks having the cells. The cell culture medium was replaced with fresh medium with or without test compounds on a weekly basis [13]. As described previously, there were no detectable effects of DMSO on cell survival, growth, and metabolic activity at the doses used in the present studies [13].

### 3.2. Cell Culture

Normal diploid human skin fibroblast cell strain, designated PCS cells, was purchased from ATCC (Catalog No: PCS-201-012 Lot No. 58732338). These cells were established from a skin biopsy from the abdomen region of a healthy 38-year-old Caucasian woman. Method for PCS cell culturing and exposure to different test compounds is described previously [13]. Briefly, PCS cells were thawed from a frozen ampoule at passage 10 (p10), and were grown in plastic tissue culture flasks with growth area 75 cm^2^ in an incubator at 37 °C, 5% CO_2_, 95% humidity, and atmospheric oxygen conditions in Dulbecco’s Modified Eagle’s Medium (DMEM; Lonza, Walkersville, MD, USA). The culture medium was supplemented with 10% fetal bovine serum (Lonza, Walkersville, MD, USA), with a mix solution of antibiotic and antimycotic agents (100 U/mL penicillin, 0.1 mg/mL streptomycin, and 0.25 mg/mL amphotericin B; Lonza, Walkersville, MD, USA). Following 95% confluency, the cells were trypsinized and distributed into new flasks at a ratio 1:2 or 1:4. This added one or two passages (p) to the culture age, respectively. In this series of experiments, serially passaged PCS cells completed a replicative lifespan of p58 taken as 100% lifespan completed. Early passage cells up to p14 were considered as young, late passage cells at p51 were considered as near-senescent, and ultimate p58 cells were considered as fully senescent. Cell numbers were monitored by using a Coulter counter, and the microscopic-photographic records of cells were kept as phase-contrast pictures either of live cells or of methanol-fixed and Giemsa stained cells, using an inverted Zeiss microscope [13].

### 3.3. Metabolic Activity, Short-Term Growth, Cell Cycle Analysis and Rejuvenation 

*MTT assay:* An assay that measures the metabolic activity and cell survival was done as described previously [13,41]. Briefly, PCS cells (p8 and p51) were seeded in 96 well plates at a density of 5000 cells per well. After 24 h of cell attachment, cells were exposed to a wide range of concentrations (0.1–100 μM) of different test compounds by replacing the old medium with fresh medium containing the test compound. The cells were incubated for 48 h, the medium was replaced with 200 μL culture medium containing 3-(4,5-Dimethyl-2-thiazolyl)-2,5-diphenyl-2H-tetrazolium bromide (MTT) reagent (0.5 mg/mL), and cells were further incubated for 4 h. Formazan crystals formed inside the cells were extracted for 15 min with 100 μL of DMSO, and the absorption was measured using a microplate reader at A595 nm with A650 nm as a reference. The data were analyzed using Microsoft Excel’s version of the Student’s T-test with two-tailed distribution with sample unequal variance for ANOVA. The results were presented as % MTT activity where the readings for the control cells were considered as 100% [13,41].

*One-step growth analysis:* Serially passaged PCS cells at p14 (early passage young cells) and p51 (late passage, near senescent cells) were seeded in 12-well plates at a density of 1 × 10^4^ cells/well. Based on the dose response data obtained from MTT assays, two doses (1 and 10 µM) of each test chemical were selected and were added 24 h after seeding the cells [13,41]. Cells were trypsinized and counted in two wells after 1, 3, 5, 7, and 9 days of treatment, while the cells in the third well were fixed in ice cold methanol followed by Giemsa staining for permanent record and photography [13,41].

*Morphological reversion/rejuvenation:* Senescent PCS cells at p58 were seeded in 6-well plates at a density of 1 × 10^4^ cells/well. One selected dose of each test chemical was added 24 h post-seeding, and the culture medium was replaced with fresh medium with or without test chemicals once a week. After two weeks the cells were methanol fixed, Giemsa stained, and microphotographed [13,41].

*Cell cycle analysis:* The cells were treated with 1 µM ABC, PTA, or SAB for 24 h, and DNA-based cell cycle analysis was performed using Muse^™^ Cell Analyzer and Muse^™^ Cell Cycle Kit according to the manufacturer’s instructions (Merck Millipore, Warsaw, Poland) [42,43].

### 3.4. Western Blotting

The method for cell sample preparation, separating proteins by polyacrylamide gel electrophoresis and transferring to blotting membranes was as described earlier [42,43]. Primary and secondary antibodies used in this study were: anti-p53 (1:1000, MA5-12557, Thermo Fisher Scientific, Waltham, MA, USA), anti-SOD1 (1:500, PA523245, Thermo Fisher Scientific, Waltham, MA, USA); anti-LC3B (1:1000, 2775, Cell Signaling Technology, Danvers, MA, USA); and horseradish peroxidase-conjugated secondary antibody (1:50000, Sigma Aldrich, Poznan, Poland). For the loading control, mouse monoclonal anti-β-actin−peroxidase antibody was used (1:40000, A3854, Sigma Aldrich, Poznan, Poland). The chemiluminescence signal was detected using a Clarity Western Blotting Detection System (BIORAD, Watford, UK) and a G:BOX imaging system (Syngene, Cambridge, UK) [31,32]. Densitometry analysis was conducted using GelQuantNET software(version 1.8.2, Biochemlabsolutions.com, University of California, San Francisco, CA, USA). Data were normalized to β-actin.

### 3.5. Protein Carbonylation and Aggregation

The levels of protein carbonylation was estimated with an OxyBlot^™^ Protein Oxidation Detection Kit (Merck Millipore, Warsaw, Poland) using the standard protocol according to the manufacturer’s instructions [43]. Protein aggregation was measured using PROTEOSTAT^®^ Protein Aggregation kit (Enzo Life Sciences, Inc., Farmingdale, NY, USA) following the standard protocol according to the manufacturer’s instructions [44]. The levels of protein aggregates are presented as relative fluorescence units (RFU). The results represent the mean ± SD from at least three independent experiments. Statistical significance was assessed by 1-way ANOVA using GraphPad Prism 5, and with the Dunnett’s multiple comparison test.

### 3.6. Real-Time PCR Using TaqMan^®^ Arrays

After exposure of early passage (p14) young and late passage (p58) near-senescent PCS cells to 1 µM ABC, PTA, or SAB treatments for 24 h, RNA was extracted and cDNA was synthesized using 2 µg of RNA as a template and Transcriptor First Strand cDNA Synthesis Kit (Roche, Warsaw, Poland) according to the manufacturer’s instructions. The expression of cyclins, cell cycle regulation-associated genes and inflammation response genes was evaluated using Applied Biosystems StepOnePlus^™^ Real-Time PCR System and dedicated Real-Time PCR TaqMan^®^ Array Plates, namely TaqMan^™^ Array, Human Cyclins and Cell Cycle Regulation, Fast 96-well (4418768, Applied Biosystems^™^) and TaqMan^™^ Array, Human Cytokine Network, Fast 96-well (4418769, Applied Biosystems^™^), respectively, according to the manufacturer’s instructions (Thermo Fisher Scientific, Waltham, MA, USA). The expression profiles were created as previously described [45]. All results were compared to control conditions (cells at p14 without treatments).

## 4. Conclusions

In conclusion, we have observed that the effects of phytochemicals can be age-related and differential in term of metabolic activity, growth rates, cell cycle regulation, oxidative damage, and inflammatory response. Our further studies are in progress to find out if such differential effects can also be observed over a life-long exposure of serially passaged human cells in vitro in terms of replicative lifespan and markers of ageing and senescence, including telomeres, DNA methylation, oxidative damage, and senescence associated secretory phenotype (SASP) and other characteristics. Another important point to emerge out of these studies is that the tested compounds also show typical biphasic dose response as reported for numerous chemicals, including phytochemicals [2,7,13]. Such studies have importance with respect to their implications for the use of such compounds as health- and ageing-interventions in terms of dosage and age.
